# Professional Inventions

**Published:** 1877-04

**Authors:** 


*Professional Inventions.*—The *Scientific American* takes issue with the *Medical Record* in regard to Physicians as Patentees, for the reason, that the new design is submitted to the instrument maker, who first takes good care to charge the designer a round sum for making it, and then goes on to manufacture and sell the article at an immense profit to himself. The *Record* takes the ground that: " First : It is the duty of medical men, to give to the world unreservedly, the benefit of all professional inventions and discoveries they may make." "Second: To take out a patent is to retain a proprietary interest in the invention patented. Therefore, it is an undignified and unprofessional thing, to patent a medical or surgical invention. But a physician may surreptitiously derive a pecuniary benefit from such an invention, or rather from the sale of it, provided he can persuade a manufacturer to allow it to him. For our part, we think that this indirect way of getting one's due, is infinitely less dignified than the straightforward matter-of-fact way provided by law.'

The *Scientific American* argues that no physician objects to the copyright of a medical book, nor does any one imagine that the dignity of the profession is in any way lowered by the circumstance, that many of its members add largely to their income by such means." " The free gift which the profession intends to make of professional inventors, thus results only in making such articles so costly as to restrict their use," and goes on to suggest a remedy as follows. " The cure of these evils hinges, we believe, on adoption of more business-like and practical views, touching this matter by the profession at large. The moment physicians and surgeons abandon their prejudice against patents, and act like other people, the business of the professional instrument maker will take on a much more satisfactory aspect. Protected in his work, he would have some inducement to improve its methods. The first result would be to cheapen the products, and so encourage their more general use. Enlarged demand would react upon the price, and that again, upon the employment of such professional aids, to the natural increase of the intelligence and efficiency of the profession. Further, invention begets invention; and whatever is done to increase the use of improved professional means and appliances, increases also the probability of still other improvements. The inventor's royalty steps in to encourage the good work, and to secure the preservation of valuable suggestions and devices, now commonly lost to the profession. The benefit that would ultimately accrue to the profession from and through this line of advancement, is quite incalculable."

